# Insight into the divergent role of TRAIL in non‐neoplastic neurological diseases

**DOI:** 10.1111/jcmm.15757

**Published:** 2020-08-22

**Authors:** Shiqi Gao, Yuanjian Fang, Sheng Tu, Huaijun Chen, Anwen Shao

**Affiliations:** ^1^ Department of Neurosurgery Second Affiliated Hospital School of Medicine Zhejiang University Hangzhou China; ^2^ State Key Laboratory for Diagnosis and Treatment of Infectious Diseases Collaborative Innovation Center for Diagnosis and Treatment of Infectious Diseases The First Affiliated Hospital College of Medicine Zhejiang University Hangzhou China

**Keywords:** apoptosis, infection, neurodegenerative disease, stroke, TRAIL, traumatic brain injury

## Abstract

Tumour necrosis factor–related apoptosis‐inducing ligand (TRAIL) is a member of the tumour necrosis factor (TNF) superfamily which mainly induces apoptosis of tumour cells and transformed cell lines with no systemic toxicity, whereas they share high sequence homology with TNF and CD95L. These unique effects of TRAIL have made it an important molecule in oncology research. However, the research on TRAIL‐related antineoplastic agents has lagged behind and has been limited by the extensive drug resistance in cancer cells. Given the several findings showing that TRAIL is involved in immune regulation and other pleiotropic biological effects in non‐malignant cells, TRAIL and its receptors have attracted widespread attention from researchers. In the central nervous system (CNS), TRAIL is highly correlated with malignant tumours such as glioma and other non‐neoplastic disorders such as acute brain injury, CNS infection and neurodegenerative disease. Many clinical and animal studies have revealed the dual roles of TRAIL in which it causes damage by inducing cell apoptosis, and confers protection by enhancing both pro‐ and non‐apoptosis effects in different neurological disorders and at different sites or stages. Its pro‐apoptotic effect produces a pro‐survival effect that cannot be underestimated. This review extensively covers in vitro and in vivo experiments and clinical studies investigating TRAIL. It also provides a summary of the current knowledge on the TRAIL signalling pathway and its involvement in pathogenesis, diagnosis and therapeutics of CNS disorders as a basis for future research.

## 1 INTRODUCTION

The tumour necrosis factor–related apoptosis‐inducing ligand (TRAIL), also known as Apo2L, was initially cloned and characterized as a new member of the TNF superfamily in the mid‐1990s.^1^, ^2^ Consisting of 281 amino acid residues, TRAIL is a type II transmembrane protein with an extracellular C‐terminal domain, which shares extracellular domain sequence (receptor‐binding motif) homology with TNF (23% identical), LTα (23% identical) and FasL/CD95L (28% identical).^1^ Similar to other highly homologous members of the TNF ligand family, TRAIL was originally regarded as a cytokine that selectively induces apoptosis in a variety of cancer cell lineages or transformed target cells,^3^ and leaving normal cells unaffected.^4^

The ability to selectively induce apoptosis in tumour cells without affecting healthy cells makes TRAIL a vital module in the field of cancer treatment,^3^ including wide utilization in the treatment of glioma in the CNS.^5^, ^6^ However, it has recently emerged that many cancers are becoming TRAIL‐resistant. This can be attributed to several TRAIL limitations which include poor agonistic activity and stability of recombinant soluble TRAIL. Cancer cells are also capable of exploiting endogenous TRAIL/TRAIL‐R system to their advantage. Many cancers have been TRAIL‐resistant, and this has made scientists explore more strategies and nanotechnological advancements to enhance the apoptosis promoting effect of trail on cancer cells.^7^, ^8^ In addition to its expression in a wide range of normal tissues, TRAIL messenger RNA is expressed in human natural killer cells, B cells, monocytes and dendritic cells following cytokine stimulation.^9^, ^10^ This means that the regulation of TRAIL‐mediated cell death is more complex than simply interacting with five distinct receptors identified and characterized previously.^11^ Therefore, in addition to anticancer effects, the pleiotropic influence of TRAIL has also been observed in various pathophysiological processes involving multiple systems, such as autoimmune diseases such as rheumatoid arthritis and systemic lupus erythematosus, cardiovascular diseases such as acute myocardial infarction and atherosclerosis.^12^, ^13^, ^14^, ^15^ Among CNS diseases, inflammation to a certain extent acts as an important link in the complex pathogenesis regulated by TRAIL. This mediates neuron damage after acute injury, promotes the formation of amyloid β plaque in Alzheimer's disease and plays a specific protective role in experimental autoimmune encephalomyelitis. Based on such a complex and ambiguous background, this paper provides an extensive review of in vitro and in vivo experimental and clinical studies, and then provide a summary of the current knowledge on the TRAIL signalling pathway and its involvement in pathogenesis, diagnosis and therapeutics during CNS disorders. The information provided here is expected to form a basis for future studies.

## 2 TRAIL SIGNALLING SYSTEM AND RELATED REGULATION DEMONSTRATED IN ONCOLOGICAL RESEARCHES

In humans, two TRAIL receptors contain a functional cytoplasmic death domain and thus can transduce the signals to induce apoptosis. They are known as membrane‐bound death receptor 4 (DR4/TRAIL‐R1) and death receptor 5 (DR5/TRAIL‐R2). These two receptors are similar in structure (58% identity) and are highly distributed in cells and tissues including peripheral blood lymphocytes (PBLs), spleen and thymus. They mediate apoptosis by associating with Fas‐associated death domain protein (FADD).^16^, ^17^, ^18^ An additional two receptors, namely glycosyl‐phosphatidylinositol (GP1)‐anchored DcR1 (TRAIL‐R3) without an intracellular domain, and DcR2 (TRAIL‐R4) containing a truncated death domain serve as decoy receptors. TRAIL can bind to DcR1 and DcR2 by their cysteine‐rich extracellular domain, but this combination cannot induce apoptosis because it lacks a functional death domain (DD). This suggests that the apoptotic capacity of TRAIL can be resisted by competitively binging to decoy receptors.^19^, ^20^, ^21^ Another unique soluble decoy receptor named osteoprotegerin (OPG), which is capable of inhibiting osteoclastogenesis in bone remodelling by interacting with OPG ligand (previously described as receptor activator of NF‐κB ligand (RANKL),^22^ also combines with TRAIL and impairs TRAIL‐induced apoptosis.^23^

**Figure 1:**
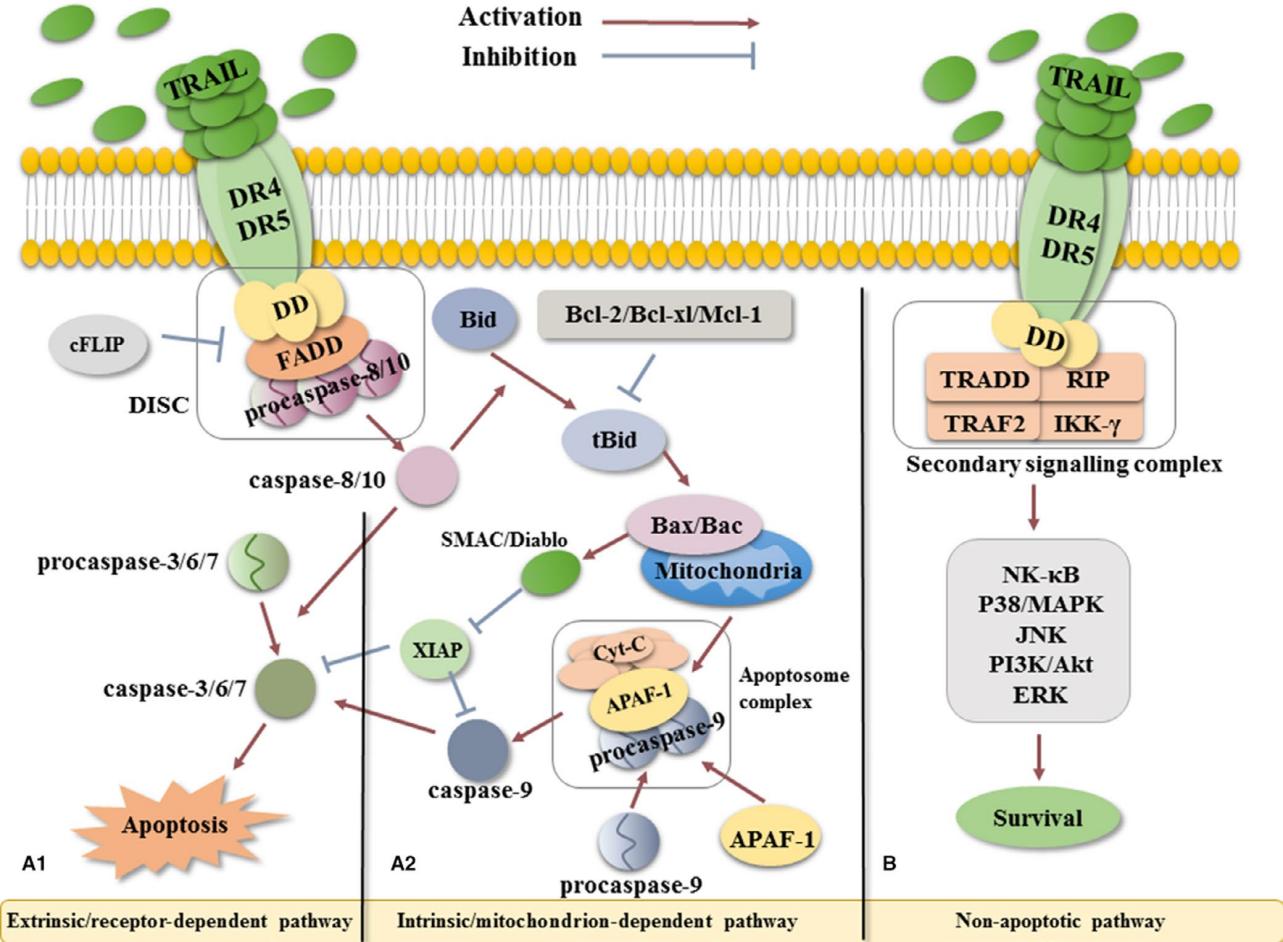
The TRAIL signalling pathway. A, Apoptotic signalling pathway. The binding of soluble TRAIL to the homotrimer of DR4/5, the DD on receptors, the recruited FADD and pro‐caspase 8 form the DISC complex, which triggers autocatalytic activation of caspase 8 in DISC. Subsequently, in (A1) extrinsic pathway, caspase 8 activates caspase 3/6/7 directly; in (A2) intrinsic pathway, caspase 8 cleaves Bid into tBid which later interact with Bax/Bac on the mitochondrial membrane leading to lysis of mitochondria. The Cyt‐C released from mitochondria cleaves pro‐caspase 9 into caspase 9 in apoptosome complex, which in turn activates caspase 3/6/7. The effector caspase 3/6/7 activated by both pathways induces cell apoptosis. B, Non‐apoptotic signalling pathway. Adaptor proteins including TRADD, TRAF2, RIP1 and IKK‐γ are recruited to form the secondary signalling complex through a serious complex processes involving several molecules such as NF‐κB, P38/MAPK, JNK, PI3K/Akt and ERK. These pathways induce cell survival, proliferation and migration among other processes. APAF‐1, apoptotic protease–activating factor‐1; Bcl‐2, Bcl‐XL, Mcl‐1, Bac, Bax all belong to Bcl‐2 family, B cell leukaemia 2 family; Bid, BH3 domain–containing protein; c‐FLIP, cellular FADD‐like IL‐1β–converting enzyme inhibitory protein; DISC, death‐inducing signalling complex; DR4/5, death receptor 4/5; ERK, extracellular regulated kinase; FADD, Fas‐associated death domain; IKK‐γ, inhibitor of κB (IκB) kinase‐γ; JNK, c‐Jun N‐terminal kinase; MAPK, mitogen‐activated protein kinases; NF‐κB, nuclear factor kappa‐light‐chain‐enhancer of activated B cells; PI3K, phosphatidylinositide 3‐kinases; RIP1, receptor‐interacting kinase 1; tBid, truncated Bid; TRADD, TNFR1‐associated death domain; TRAF2, TNF receptor–associated factor 2; TRAIL, TNF‐related apoptosis‐inducing ligand; TRAIL‐R, TNF‐related apoptosis‐inducing ligand receptor; XIAP, X‐linked inhibitor of apoptosis protein

As described above, TRAIL facilitates pro‐apoptotic and non‐apoptotic effects by binding to death or decoy receptors. The apoptosis course can be triggered by either intrinsic or extrinsic pathways initiated from the formation of a homotrimer of DR4 or DR5.^24^ This homotrimer subsequently recruits FADD and pro‐caspase 8/10 to form the death‐inducing signalling complex (DISC).^25^ Cellular FADD‐like IL‐1β–converting enzyme inhibitory protein (c‐FLIP) can also be recruited to DISC as an inhibitor of caspase cascade via heterodimerizing with pro‐caspase 8.^26^ In this multi‐protein complex of DISC, pro‐caspase 8/10 is cleaved and activated autocatalytically, producing a vigorous proteinase caspase 8/10 which can cleave multiple downstream proteins such as pro‐caspases 3, 6 and 7, and BH3 interacting domain death agonist (Bid).^27^, ^28^ Recently, according to the ratio of XIAP to caspase 3 and the DISC’s capacity to cleave pro‐caspase 3, cells were classified into type I and type II.^29^, ^30^ On the one hand, type I cells activate caspase 8/10 which are sufficient or more than enough to cleave pro‐caspase 3 and then trigger apoptosis directly, and this pathway is known as an extrinsic apoptotic pathway or receptor‐dependent pathway ^30^, ^31^, ^32^ (Figure 1A1). On the other hand, type II cells activate caspase 8/10 converting Bid into truncated Bid (tBid) which later interacts with Bak and Bax proteins on the membrane of mitochondria and leads to the release of cytochrome c and SMAC/Diablo from the unstable mitochondria outer membrane. Cytochrome c couples with pro‐caspase 9 and apoptotic protease‐activating factor‐1 (APAF‐1) and assembles into apoptosome complex, which sequentially activates caspase 9 and executioner caspase 3. The SMAC/Diablo also promotes apoptosis as it serves as the negative effect on XIAP, which is a direct inhibitor of final effective apparatus, caspase 3/9. The effect of SMAC/Diablo might be crucial in case of insufficient caspase 8. This pathway is known as an intrinsic apoptotic or mitochondrion‐dependent pathway^30^, ^31^, ^32^ (Figure 1A2).

Apart from classic pathways, TRAIL can also participate in the non‐apoptotic pathway in certain situations (high concentrations of TRAIL). This non‐canonical signalling can lead to cell survival, proliferation and migration via transcription of several genes at the stimulation of kinase signallings, such as nuclear factor kappa‐light‐chain‐enhancer of activated B cells (NF‐κB), mitogen‐activated protein kinases (P38/MAPK), c‐Jun N‐terminal kinase (JNK), phosphatidylinositide 3‐kinases (PI3K/Akt) and extracellular regulated kinase (ERK).^33^, ^34^ For instance, NF‐κB, a familiar transcription factor, could up‐regulate the transcription of genes such as c‐FLIP, Bcl‐XL and XIAP. These genes encode intracellular cytokines that are capable of blocking specific sites during the apoptotic processes as explained earlier, thus inhibits apoptosis.^35^ The P38/MAPK as a multifunctional kinase could regulate inflammation, cell proliferation and differentiation as well as apoptosis.^36^ The ERK protects the integrity of the endothelial cell and promotes proliferation.^37^ The PI3K‐independent activation of protein kinase B/Akt plays a crucial role not only in cell survival triggered by growth factors, extracellular matrix and other stimuli but also in activation of NF‐kB pathway^38^ (Figure 1B).

TRAIL selectively driven apoptosis of tumour cells has been extensively studied in the field of cancer treatment as it was found in the mid‐1990s. However, one of the biggest barriers to its effective clinical application lies in the drug resistance against TRAIL‐based therapeutics. Among various reasons including decline in of death receptors, activation of oncogenes and silence of tumour suppressor genes, and disorder of anti‐apoptotic proteins, the dark side of TRAIL‐mediated signalling which also known as non‐canonical survival signalling pathways has achieved more attention than TRAIL‐induced apoptotic signalling.^39^, ^40^ Additionally, according to the cellular context, TRAIL signalling towards death or survival may be affected by factors such as variation between the expression of death receptors and decoy receptors, shuttling of death receptors and regulation of non‐coding RNA.^39^, ^40^ In a large amount of cancer treatment studies, these factors were reported to confer TRAIL‐based therapeutics potential adverse effect such as proliferation, migration and metastasis of cancer cells. For example, studies on breast cancer metastasis reported that overexpressed DR5 was able to improve the expression of C‐X‐C chemokine receptor type 4 (CXCR4) on the surface of MDA‐MB‐231 BCa cells, whose migratory potential towards stromal cell–derived factor 1 (SDF1) was subsequently enhanced.^41^ Interestingly, a natural agent a‐mangostin was found to significantly promote shuttling of nuclear accumulation of DR5 to cell surface to break the suppressed condition of TRAIL‐driven apoptosis.^39^ Moreover, a‐mangostin can also positively regulate TRAIL‐mediated apoptosis by significantly enhancing the expression of DR5 through down‐regulating miR‐133b.^39^ Actually, TRAIL pathways were demonstrated to be regulated by abundant non‐coding RNAs such as long non‐coding RNA (lncRNA) and microRNA (miRNA), in which miRNA was most widely studied.^42^ Non‐coding RNAs possess a bright prospect in the field of TRAIL‐based tumour therapy and drug resistance relief because most of which positively or negatively regulate the components of TRAIL/TRAIL‐R system.^39^, ^42^ Unfortunately, there are very limited researches about non‐cording RNA on CNS diseases up to now. The study of TRAIL in CNS diseases still stays in the classical apoptotic pathway, and even the dark side has not gain enough attention, let alone non‐coding RNAs as the regulators. It reminds us that many valuable and novel experiences can be drawn from the field of tumour researches for the study of TRAIL in CNS.

## 3 TRAIL AND NON‐NEOPLASTIC NEUROLOGICAL DISEASES

### 3.1 Acute brain injury

#### 3.1.1 The role of TRAIL in ischaemic stroke and haemorrhagic stroke

Cerebral stroke, one of the fatal and disabling diseases worldwide, which cause brain tissue damage because of the sudden rupture of blood vessels or the inability of blood flow because of blockage of blood vessels in the brain, includes haemorrhagic stroke and ischaemic stroke.^43^ There are certain research foundations of TRAIL on ischaemic stroke, whereas no literature about haemorrhagic stroke and TRAIL can be retrieved in PubMed. Thus, in this section, we mainly discuss the progress of TRAIL/TRAIL‐R system in ischaemic stroke, and our unpublished data which showed the relationship between TRAIL and haemorrhagic stroke will be discussed in the discussion section. Accounting for the majority of stroke events, ischaemic stroke shows various sudden clinical symptoms such as lacunar‐like syndromes, aphasia or neglect, ataxic syndromes, visual field defects and associated neurobehavioural syndromes which mainly based on occlusion of different blood vessels.^44^ Ischaemic stroke causes tissue impairment not only through direct damage such as oxygen and glucose deprivation or increased intracranial pressure but also through secondary inflammatory response followed by cell death. After the onset of ischaemic stroke, the hypoxia‐ischaemia area suffers from fatal cerebral swelling, and later releases tremendous amounts of inflammatory mediators and damage‐associated molecular patterns (DAMPs).^45^ This is usually followed by prominent infiltration of immune cells.^45^ The TRAIL protein is not found in normal brain tissue,^46^ but TRAIL is showed to participate in the neuronal apoptosis through a ceramide‐mediated c‐Jun signalling pathway after brain injury.^47^, ^48^ Recruitment of leucocytes by crossing the compromised blood‐brain barrier (BBB) and activation of local microglia seem to play a crucial role in the initiation of neuronal apoptosis.^49^, ^50^, ^51^ Several pre‐clinical studies indicate that TRAIL is mainly expressed in active microglia and macrophages, whereas DR5 is predominantly expressed in neurons after transient global cerebral ischaemia.^52^ Meanwhile, the competitive inhibitor‐soluble DR5 can reduce the cerebral pathological harm by competitively binding to free TRAIL.^52^ Similar changes in TRAIL and its receptor are also found in hypoxia‐ischaemia (HI) of immature rat models.^50^, ^53^ In these studies, however, DcR1 was overexpressed in the cerebral cortex.^50^

Accounting for approximately 85% of all stroke cases,^54^ ischaemic cerebral stroke has attracted much attention. However, many agents that are effective in pre‐clinical studies have not suitable for clinical application.^55^ Because of this, some researchers have provided more information on an endogenous neuroprotective mechanism, for example ischaemic pre‐conditioning (IPC),^56^ which causes an effect by regulation of intrinsic TRAIL and its receptors rather than extrinsic agents. The IPC conferred tolerance to ischaemic neurons when rats were subjected to sublethal brain ischaemia in 30 mins of tMCAO study as reported by Cantarella et al.^49^ The TRAIL and its death receptors were down‐regulated, whereas decoy receptors were up‐regulated after IPC, and this led to the suppression of intrinsic and extrinsic apoptotic pathways. Recently, Xu et al reported that remote limb pre‐conditioning (RPC) ameliorates brain damage after ischaemic cerebral stroke with similar regulation of TRAIL.^54^ As an oxygen‐free radical scavenger, edaravone provides neuronal protection against hypoxic‐ischaemic brain damage by suppressing TRAIL and active caspase 3 protein.^57^ Intriguingly, previous studies have demonstrated that the condition of oxidative stress characterized by excessive accumulation of reactive oxygen species mediates the overexpression of DR5 through key downstream transcription factors such as C/EBP‐homologous protein (CHOP).^58^ On the basis of this theory, various modulators, inducers and sensitizer of ROS were researched to enhance the sensitivity of TRAIL in a lot kinds of cancer cells, which is a promising research target towards TRAIL‐related tumour drug resistance.^59^, ^60^ Conversely, in most cases, the primary task of the TRAIL‐based therapeutics for CNS diseases is inhibiting the apoptotic pathway. Therefore, exploring an effective ROS inhibitor may be a novel and promising target compared with the traditional direct inhibition on TRAIL or its death receptors. Utilization of edaravone on hypoxic‐ischaemic brain damage filled this research blank and created a precedent.

Coalition pre‐conditions mentioned above with the application of TRAIL signalling pathway inhibitors may provide a comprehensive approach to ischaemic cerebral stroke (ICS) treatment.

The TRAIL and its receptors have been recognized as biomarkers in various diseases (Table 1). There were significant negative correlations between the level of TRAIL in serum and stroke scale score and volume, demonstrated by clinical research in acute ischaemic stroke. Among different stroke subtypes, serum TRAIL levels had no obvious distinction.^61^ Interestingly, plasma levels of TRAIL‐R5 (OPG) significantly increased along with the presence and severity of cerebral atherosclerosis. These results were supported by Pan et al who revealed that TRAIL‐R2 (DR5) displays similar changes in large artery atherosclerosis (LAA) stroke.^15^ Recently, a study conducted in a cohort of 95 first‐ever acute ischaemic stroke patients and 95 healthy controls reported that there was a change in TRAIL levels in the peripheral circulatory system after the onset of stroke events. Serum TRAIL protein levels were increased, whereas mRNA expressed on peripheral blood mononuclear cells (PBMC) was down‐regulated as the condition progressed. This shows that TRAIL accord with the chronic regulatory processes from transcription to translation and therefore promises to be a valuable biomarker of ICS.^62^

**Table 1:** A list of clinical cohort researches of sTRAIL or TRAIL mRNA as well as other relative factors as biomarkers in CNS non‐neoplastic diseases

Disease	Patients	sTRAIL changes	TRAIL mRNA changes	Main findings	Remark	Year/Ref.
ICS	95 first‐ever patients and 95 HC	Lower levels at the onset; ↑ later periods	↑ at the onset; ↓in later periods	Reliable predictor of stroke outcome	/	2018/^62^
	293 patients	(‐) NIHSS score and stroke volume within 7 days of onset	/	Associated with ICS severity	(∓) stroke subtypes	2015/^61^
SCI	22 chronic SCI and 19 HC	Detectable levels during chronic SCI	/	Potential biomarker or treatment site	Supplement for MIF as a biomarker	2013/^124^
TBI	10 mild and 10 severe TBI patients and 10 controls	↑↑ within the first hour of TBI	/	Able to discriminate between TBI and HC at < 1 h	Cooperate with AXIN1 as biomarkers	2017/^70^
AD	103 older individuals with memory concerns without cognitive impairment	(+) annual rate of change in episodic and working memory	/	Bone‐related biomarkers may predict worsening cognition	Dkk1 (+) semantic memory; Dkk1 (‐) working memory	2018/^125^
	22 AD patients and 20 HC	(‐) MMSE scores in AD patients; (∓) AD and HC	(∓) in the PBMCs of AD and HC	Down‐regulate the peripheral immune response in the late stages	no TRAIL protein in the CSF samples	2009/^94^
MS	53 MS patients and 25 HC	Lower in MS patients	(∓) MS patients	Apoptosis of T cells ↓ in MS patients		2014/^126^
	92 untreated MS patients and 38 HC	↓↓during relapses of RRMS	/	Participate in the pathogenesis of MS	sFas, sFasL and sTRAIL (∓) MS patients and HC	2013/^118^
	35 RRMS patients and 19 HC	↑ during IFN‐β treatment	/	Indicating the response to IFN‐β therapy at individual level	↓ surface TRAIL expression on lymphocytes	2011/^127^
	26 HC and 21 SPMS, 70 RRMS	↑in relapse of untreated MS patients; ↑in remission of IFN‐β–treated patients	↑in remission of IFN‐β treated patients	Association with MS disease activity; Reflecting bioactivity of IFN‐β	/	2008/^128^
	15 HC and 15 untreated RRMS, 9 glatiramer acetate‐treated and 37 IFN‐β–treated RRMS patients	Related to subjective flu‐like symptoms observed	/	Unable to predict the therapeutic response in long‐term IFN‐β–treated patients	/	2007/^129^
	30 HC and 73 RRMS patients	/	22% abrogation of TRAIL expression after long‐term INF‐β treatment	MxA as an appropriate biomarker for IFN‐β	MxA expression was higher than both TRAIL and XAF‐1	2006/^130^
	82 MS patients	Before treatment, initial, sustained ↑ with better INF‐β treatment response	/	Prognostic marker of treatment response to INF‐β	/	2003/^110^
	19 MS patients and 14 controls during late pregnancy and post‐partum	↑ from late pregnancy to post‐partum situation in both MS patients and controls	/	TRAIL may increase the risk of relapses in MS post‐partum	/	2010/^131^

Abbreviation: (‐), inversely correlate with; (+), positively correlate with; (∓), no significant differences between; ↑, up‐regulate; ↓, down‐regulate; AD, Alzheimer disease; AXIN1, axis inhibition protein 1; CSF, cerebrospinal fluid; Dkk1, Dickkopf WNT signalling pathway inhibitor 1; HC, health control; ICS, ischaemic cerebral stroke; MIF, migration inhibitory factor; MMSE, Mini‐Mental State Examination; MS, multiple sclerosis; MxA, myxovirus resistance protein A; NIHSS, National Institute of Health Stroke Scale; PBMCs, peripheral blood mononuclear cells; RRMS, relapsing‐remitting multiple sclerosis; SCI, spinal cord injury; SPMS, secondary progressive MS; TBI, traumatic brain injury; XAF‐1, X‐linked inhibitor of apoptosis factor‐1.

#### 3.1.2 The role of TRAIL in traumatic brain and spinal cord injuries

Traumatic brain injury (TBI) and spinal cord injury (SCI) are life‐threatening conditions that mainly result from trauma caused by motor vehicle accidents or violence on two different neurological sites.^63^, ^64^ In the United States, there are around 85 per 100 000 people are hospitalized and over $60 billion was costed for TBI every year. For SCI, the incidence was reported to lie between 10.4 and 83 per million residents per year, and one‐third of patients are tetraplegic, whereas 50% of patients have a complete lesion. These two diseases mainly occur in young people and are usually accompanied by severe paralysis, which increases the burden on society.^63^, ^64^ The exact pathophysiological mechanism of these two disorders is quite complicated and interconnected. They can, however, be roughly categorized into two stages: primary injury characterized by shearing, tearing or stretching of nerves and their surrounding tissues,^65^ and secondary injury characterized by various processes including ionic dysregulation, neurotransmitter accumulation, neuronal apoptosis and immune‐associated neurotoxicity.^66^, ^67^ Because the primary mechanical injury is usually instantaneous and unpredictable, it is hard to adopt timely intervention measures. On the contrary, the secondary injury‐induced neurological damage shows a delay phase; therefore, accurately identifying the stage and severity of TBI and SCI is crucial for optimizing the treatment to avoid neurological function impairment.^68^, ^69^ Lisa et al recently determined the value of TRAIL along with AXIN1 as potential serum biomarkers in discriminating between TBI and healthy volunteers within the first hour. These results may provide a pre‐hospital first aid team with a basis for decision‐making during the treatment of TBI patients.^70^

Moreover, it has been discovered that TRAIL is one of the various overexpressed inflammatory mediators after spinal cord injury and the expression is induced not only by intraspinal injection of quisqualic acid (QUIS) but also by clip compression injury persisting for 30 minutes. The pathophysiological characteristic of QUIS is similar to traumatic SCI, and the peak of TRAIL mRNA expression levels was observed at the 1‐ to 2‐week time‐point following QUIS injury.^71^ A relatively early expression of TRAIL was observed in another study. High‐intensity TRAIL protein was inclined to colocalize with oxygenase‐1 (HO‐1) in motor neurons after 16 hour post‐operation.^72^

Cantarella et al were the first to highlight the involvement of TRAIL in the inflammatory response and cellular apoptosis after SCI. In agreement with several findings concluded from SCI models, the processes mentioned above could be halted by the TRAIL‐neutralizing antibody, therefore reduce apoptotic cells and promote neurological recovery. There is a synergizing function between glucocorticoid‐induced TNF receptor superfamily‐related ligand (GITRL) relative pathway and TRAIL pathway in the process of SCI‐related apoptosis.^73^

### 3.2 The role of TRAIL in central nervous system infection

The incidence of infectious diseases of CNS including bacterial meningitis, viral meningoencephalitis, tuberculous meningitis and cerebral malaria is still high in developing countries because of poor sanitation. Even in America with relatively hygienic living conditions, there are still more than 20,000 new patients of encephalitis or meningitis, of which viruses are the major causative agents in line with the rest of the world. Bacterial meningitis such as meningococcal disease tends to epidemic outbreak, especially in Africa. High mortality and permanent neurological sequelae in most survivors result in a heavy social burden.^74^, ^75^, ^76^ More focus and financial resources should, therefore, be channelled in the identification of exact pathogenesis and therapeutic interventions. Usually, some specific pathogens cause disruption of BBB in various ways and manage to enter CNS. To eliminate these highly virulent and neuroinvasive pathogens and reduce their reproduction, a series of cytokines are activated to stimulate innate and adaptive immune responses. Nevertheless, the excessive immune response often results in local microenvironment destruction and neurological damage which manifests by causing serious complications.^74^, ^77^, ^78^

As a classical model for studying mechanisms of injury caused by a viral infection, the reoviruses‐infected mouse study reveals that TRAIL released from infectious cells plays a crucial role in the apoptosis of neurons in vivo with neurotropic viral infection by interacting with death receptors.^79^ An experiment on high‐neurovirulence GDVII virus complements the above study by concluding that overexpressed TRAIL induces astrocytes apoptosis in murine without an increase in DR4 or DR5, but with up‐regulation of TNF‐R.^80^ Other studies that focused on CNS opportunistic infections of HIV‐1 have demonstrated the co‐location of TRAIL protein and HIV‐1–infected macrophages. Large amounts of TRAIL produced by activated macrophages indirectly mediate neurotoxicity and eventually result in HIV‐associated dementia manifested as behavioural abnormalities, motor dysfunction and so on.^81^, ^82^ Recently, TRAIL was found to induce subsequent widespread apoptosis initiated from brain endothelial cells (BECs) infected with wild‐type measles virus even with a small number.^83^

Previous studies focused on searching for potential therapeutic interventions by blocking TRAIL‐induced neurological damage after CNS infection. For instance, Kaul et al found that TRAIL released from CD8^+^ T cells protects neurons in CNS from the West Nile virus (WNV) infection to a certain extent.^84^ Similar immunomodulatory and neuroprotective effects have been demonstrated in a bacterial meningitis model. The TRAIL‐deficient meningitis model showed severe apoptosis in the hippocampus and experienced drawn‐out acute inflammation, which was moderated by intrathecal application of rTRAIL.^85^ These illustrations suggest that the challenge of investigating and developing TRAIL‐related agents lies in its complicated dual effects of inducing neuronal apoptosis and eliminating pathogens in the presence of invasive infections in CNS.

### 3.3 TRAIL and neurodegenerative disease

#### 3.3.1 The role of TRAIL in Alzheimer's disease

Alzheimer's disease (AD) is a progressive neurodegenerative disorder resulting from complicated interactions among multiple factors that progressively lead to a deficiency in neurons and the decline of cognitive functions.^86^ With the aetiology still remaining unknown, dementia is clinically characterized by memory disorder, aphasia, loss of use, loss of recognition, impairment of visuospatial skills, executive dysfunction and personality and behaviour changes. With increasing life expectancy, AD is advance irresistibly prevalent all over the world. In present America, more than 5 million residents were affected by AD and related financial burden reached $230 billion. These two numbers were excepted to reach 13.8 million patients and $1.1 trillion by 2050.^86^ Against the backdrop of increasing ageing population worldwide, multi‐filed and interdisciplinary theories and approaches have been applied in clinical research with regard to pathogenesis, early biomarkers and novel therapeutic interventions.^87^, ^88^

Several studies have confirmed that amyloid‐β plaques and neurofibrillary tangles are the two main pathological changes during AD. Nevertheless, recently, third core pathogenesis known as microglial‐related inflammation which contributes to driving and exacerbating AD pathology has been accepted as another central mechanism.^86^, ^88^, ^89^ Before the involvement of TRAIL in β‐amyloid protein (βAP), induced neurotoxic effects were demonstrated by in vitro SH‐SY5Y neuroblastoma models.^90^ Plenty of evidence has proved that the c‐Jun N‐terminal kinase (JNK) pathway, one of the TRAIL‐induced non‐apoptotic signalling pathways, contributes to βAP‐induced death of cortical neurons by induction of apoptosis‐related gene transcription.^90^, ^91^ Moreover, TRAIL is highly overexpressed in AD patients in vivo, whereas its expression has not been detected in the brains of the age‐matched non‐demented patients. And TRAIL protein tends to be detected in AD–affected neuron‐rich cerebral cortex, mainly in the proximity of amyloid plaques.^92^ Another crucial factor is that DR5 only increases under the condition of the highest toxic dose of βAP in the AD brain, and TRAIL expression shows dose‐dependent with βAP. The blockade of the DR5 significantly prevents β‐amyloid neurotoxicity both in neuronal cell line and in cortical neurons treated with βAP1‐42.^91^, ^93^

During the early stages of AD when amyloid plaques and neurodegeneration have not widely spread, the identification of AD and treatment timely seems to be the most effective approach.^87^ However, TRAIL protein could not be found in the cerebrospinal fluid (CSF) of AD patients in a study, and the serum concentration of TRAIL protein showed no difference between AD patients and normal controls.^94^ This pattern of distribution suggests it may be difficult to develop TRAIL as an early simple biomarker efficiently and accurately identifying the severity of the amyloid‐β plaques. More recently, an experiment carried out on patients with different types of dementia and mild cognitive impairment (MCI) demonstrated that soluble‐TRAIL levels in serum were higher in vascular dementia (VAD), 'mixed' dementia (MIX) and MCI patients, compared with late‐onset AD (LOAD) patients and the controls. And the discrepancy of TRAIL levels in serum may show a positive correlation with the degree of vascular damage.^95^

Apart from neurons, oligomeric Aβ may also be deposited on arteries, arterioles and capillary vascular endothelium. This leads to focal ischaemia, alterations in cerebral blood flow and cerebral micro‐/macro‐haemorrhages through potential extrinsic apoptosis on endothelial cells mediated by DR4 and DR5.^96^ In contrast to the up‐regulation of DR4 and DR5, the down‐regulation of c‐FLIP, a critical inhibitor of caspase 8 in the apoptosis signalling pathway, suggests a possibly effective therapeutic strategy aimed at increasing c‐FLIP expression in neurons. It inhibits both TRAIL‐induced and Fas‐induced apoptosis in a variety of neurodegenerative diseases.^97^ Despite the earlier belief that neutralizing TRAIL protein by antibodies that block the initiation of TRAIL signals is a risky approach because TRAIL plays a crucial role in immune surveillance,^96^ an experiment on the triple transgenic mouse model of Alzheimer's disease applying this method of neutralization of TRAIL achieved notable results.^98^ Giuseppina et al reported that when they treated their AD model with monoclonal antibody of TRAIL, they observed microscopic pathological improvement which includes reduced expression of TRAIL itself, its receptors and amyloid‐β, mitigation of inflammation and reduction in neurofibrillary tangles and macroscopic improvement of cognitive function.^98^

#### 3.3.2 The role of TRAIL in multiple sclerosis

Multiple sclerosis (MS) is a chronic autoimmune–demyelinating disease occurring in the central nervous system. Over 2 million people affected by this incurable disease worldwide. In the United States, around 400,000 patients cause approximately $10 billion economic burden annually. The disease mainly affects young adults and manifests as dissemination of lesion in space (including white matter, grey matter, brain stem, spinal cord and optic nerve) and time (the potential result of an unscheduled inflammatory invasion of CNS).^99^, ^100^ A series of progressive symptoms such as limb weakness and paresthesia, monocular visual loss, one and a half syndrome and ataxia all impose significant impacts on the physical, mental health and financial status of a patient.^100^ Many scientists believed that innate immunity is characterized by the pro‐inflammatory effect of activated macrophages and microglia, and adaptive immunity marked by cytotoxicity induced by helper (CD4+) and cytotoxic (CD8+) T cells is primarily responsible for pathological changes such as demyelination, axonal or neuronal loss, and astrocytic gliosis.^101^ The effect of B cells has also attracted renewed attention because of its role in the induction of T cells and the success of B cell–depleting antibodies in limiting pathological and clinical manifestations of MS.^99^, ^100^, ^102^

Two crucial functions of TRAIL signalling system, that is modulation of immune function and induction of apoptosis, have been confirmed by several studies and pre‐clinical applications in investigations of MS. Even if during expression of death receptors (DR4 and DR5), antigen‐specific human T cells can be exempted from apoptosis by the extracorporeal condition of soluble TRAIL, TRAIL merely inhibits activation of T cells by negatively regulating calcium influx and blocking cell‐cycle progression.^103^, ^104^ This deficiency in apoptotic elimination of autoreactive T cells may be a potential cause of MS. But, TRAIL expressing from neuroinflammatory T cells induces apoptosis of brain cells in epilepsy.^105^ Intracerebral TRAIL also critically contributes to irreversible CNS neuron apoptosis within the autoimmune inflamed brain as demonstrated in a murine MS model named experimental autoimmune encephalomyelitis (EAE).^106^ This is the most common and currently used experimental model for the pathogenesis studies of MS, in addition to, testing or developing novel agents of MS. Although it is challenging to accurately simulate the progress of MS, this model has been successful in revealing the mechanisms.^107^ In this model, however, mice treated with intraperitoneal injection of sDR5 that competitively blocks TRAIL exacerbated EAE, as demonstrated by higher disease scores and degree of inflammation in the CNS.^108^, ^109^ These diametrically opposed functions of TRAIL in the same model may be because of differences in sites of action of TRAIL and different stages of EAE.

Interferon‐beta (IFN‐β) is the main immunomodulatory agent for relapsing‐remitting MS (RRMS) through the mechanism of inhibition of T cell proliferation and blocking of blood‐brain barrier opening.^110^ And because of the instability of efficacy in different patients, researchers are seeking to find an exact biomarker for IFN‐β treatment. Wandinger et al were the first to propose that the TRAIL had the potential to become the prognostic marker of treatment response to IFN‐β in MS. This proposal was supported by the positive correlation between pre‐treatment assessment of TRAIL expression and prognosis after IFN‐β treatment.^110^ Contrarily, IFN‐β enhances TRAIL’s inhibitory effect on T cell activation as well as its expression in vivo and in vitro.^111^ Although a recent study drew a similar conclusion regarding the correlation between long‐term IFN‐β treatment and the expression of splice variants of TRAIL and its receptor in diverse cellular subsets,^112^ the controversy over this correlation has persisted in many studies over the years. This suggests that larger sample size studies are necessary for the future. Another controversy is the relationship between genetic polymorphism in TRAIL including its receptor and susceptibility to MS. In clinical studies conducted in populations of different countries, results showed no statistical correlation.^113^, ^114^, ^115^ Except for the presence of the CC genotype at position 1595 in exon 5 which represented a higher risk of MS, and the combination of the alleles G/T/ A in single‐nucleotide polymorphisms (SNPs) was shown to reduce risk of MS.^116^, ^117^

Based on the above‐mentioned studies, TRAIL has received much focus because of its potential role in the pathogenesis of MS, which has been supported by two independent studies. The first shows that sTRAIL levels significantly decrease in relapses stage of relapsing‐remitting multiple sclerosis (RRMS) with no reduction in serum levels between MS patients and healthy control.^118^ The second, however, reported that the sTRAIL level was lower in MS patients compared with the controls in the context of undifferentiated TRAIL mRNA transcription. Significant progress has also been made at the TRAIL‐related pre‐clinical treatment of MS. By intrathecal delivering of a plasmid DNA coding for fusion protein (OX40‐TRAIL) composed of a tumour necrosis factor receptor and TRAIL, the EAE mouse significantly showed a decrease in the severity of clinical disease resulting from overexpression of OX40‐TRAIL in CNS.^119^ A more promising agent with a similar mechanism, that is, fusion protein named Fn14·TRAIL which is composed of extracellular domains of Fn14 (capable of blocking the pro‐inflammatory TWEAK ligand) and TRAIL, strongly inhibits the clinical course of EAE by its anti‐inflammatory effect on various processes of CNS.^120^

Demyelination induced by oligodendrocyte death is a characteristic pathological change in MS. Several reports have revealed that the p53‐TRAIL‐JNK pathway is involved in the injury of oligodendrocyte.^121^, ^122^ Although it has been proposed that TRAIL with its decoy receptors may protect oligodendrocyte from apoptosis, a more recent study based on EAE models confirmed that TRAIL significantly induces apoptosis of oligodendrocyte in white matter by increasing the accumulation of MBP antibodies.^123^

## 4 CONCLUSION AND PERSPECTIVE

**Figure 2:**
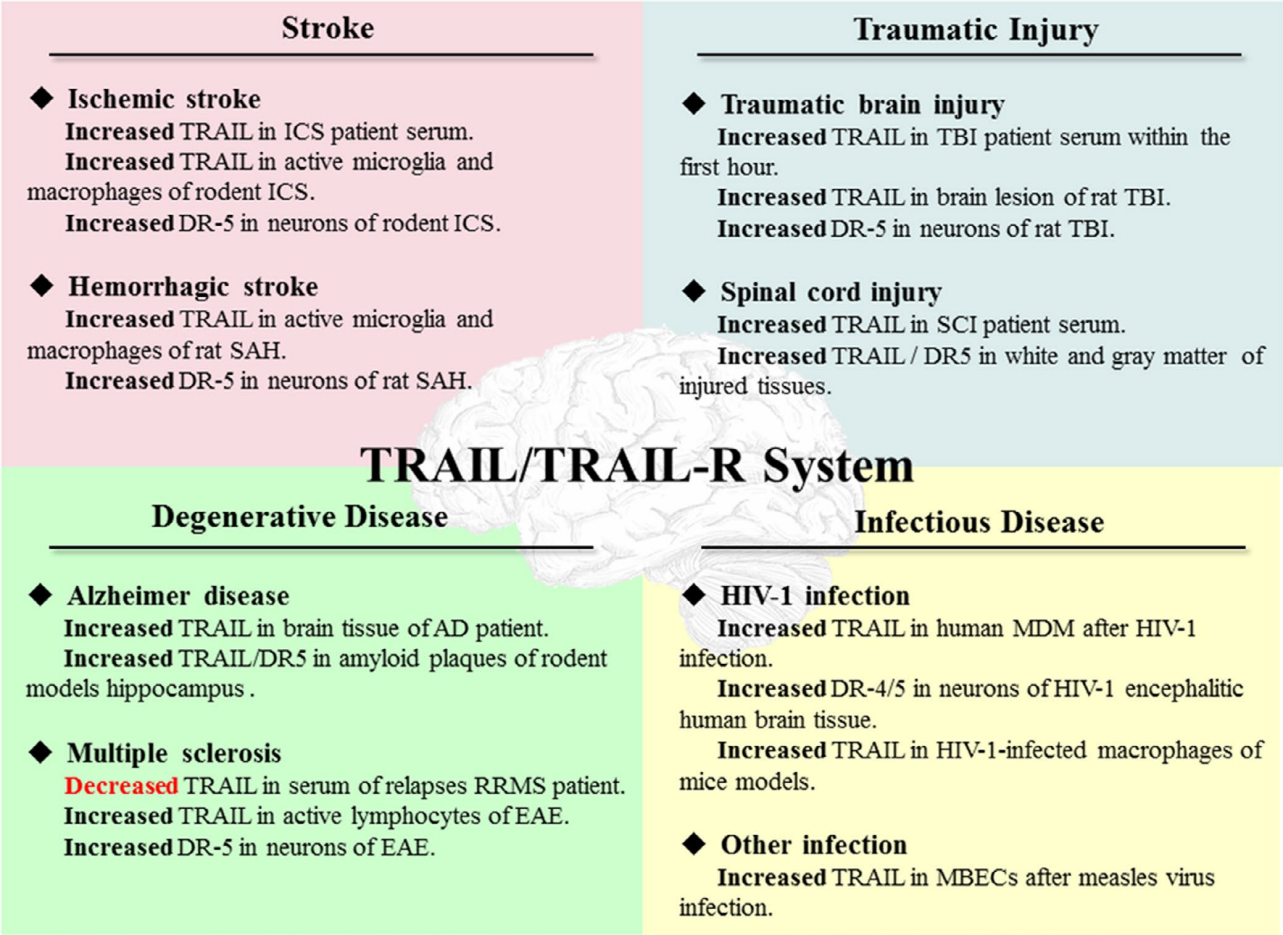
The changes in TRAIL/TRAIL‐R system in the non‐neoplastic neurological diseases. MBECs, murine brain endothelial cells; MDM, monocyte‐derived macrophages; RRMS, relapsing‐remitting MS

TRAIL, a signalling protein expressed during specific pathological conditions, plays a multifaceted role in various non‐neoplastic neurological diseases mentioned above (Figure 2). Actually, by mediating cell apoptosis, TRAIL mainly causes tissue damage accompanied by irreversible neurological damage in most cases. With a few exceptions in CNS infection of HIV‐1 and MS, TRAIL may protect neurons from autoimmune reactions (Table 2). This dual role of TRAIL suggests that elucidating specific pathophysiological changes and clarifying specific tissues or cells where TRAIL is produced as well as the downstream pathways activated by TRAIL is crucial. This concept is in line with our two unpublished studies about TBI and SAH, in which DR5 in neurons as well as TRAIL in macrophages and microglia is significantly increased after the event TBI and SAH. As for the DcR1,2‐regulated non‐apoptotic pathway, except for the positive role benefiting from up‐regulated decay receptors after IPC, there are no other positive results of pre‐clinical experiment so far. Indeed, therapeutics based on TRAIL inhibition or death receptors have achieved considerable results. Especially in the field of cancer therapy, even with widespread drug resistance, scientists are committed to finding efficient solutions and several TRAIL‐based therapeutics assisted by sensitizers, miRNA like miR‐34a and efficient delivery methods which have been in different stages of clinical trials. Researches of TRAIL on non‐neoplastic neurological diseases can be regarded as a reasonable expansion of the original field of tumour treatment, but the difference is that we need to inhibit normal CNS cell apoptosis as much as possible. Inhibiting TRAIL‐induced apoptotic signalling pathway via exploring efficient inhibitors, neutralizers and antagonists of TRAIL or death receptors is the people's initial thinking about TRAIL in CNS. It is undeniable that these thoughts have achieved certain transformation; however, more novel perspectives are worth our reference. For example, ROS was demonstrated to up‐regulate the expression of DR5 as mentioned above. Thus, inhibiting TRAIL‐induced apoptotic signalling through down‐regulating DR5 expression by application of free radical scavenger may be a promising target. Similarly, the expression of TRAIL and TRAIL‐R can also be regulated by various endogenous substances such as non‐cording RNA, especially miRNA. Different non‐cording RNA can positively or negatively regulate TRAIL/TRAIL‐R expression. As positive regulation of miRNA for TRAIL‐driven apoptosis in cancer therapeutics researches has made great progress, negative regulation may also gain a reasonable hypothesis in CNS anti‐apoptotic treatment.

**Table 2:** Summary of TRAIL/TRAIL‐R system's dual role as well as relevant treatment which utilize or restrain this system in various models of CNS diseases

Animal models	Changes in TRAIL/TRAIL‐R	Effects of TRAIL/TRAIL‐R	Treatment	Main findings	Ref
TRAIL/TRAIL‐R system play as a damage role					
BCCA occlusion–induced ICS	↑ TRAIL in A‐MI/MA; ↑ DR5 in neurons	Induce neuron apoptosis	sDR5	Reduced delayed neuronal damage	^52^
Clip compression model of SCI	↑ TRAIL and ↑ DR5 in white and grey matter injured tissues	Induced apoptosis	TRAIL‐neutralizing antibody	Prevented SCI‐induced apoptosis; improved motor function	^73^
CNS HIV‐1–infected mice model	↑ TRAIL in HIV‐1–infected macrophages	Induce neuron apoptosis	TRAIL‐neutralizing antibody	Blocked the neuronal apoptosis	^81^
3 × Tg‐AD mice model	↑ TRAIL and DR5 in the hippocampus	Mediated amyloid neurotoxicity	TRAIL‐neutralizing antibody	Improvement of cognitive parameters	.^98^
Encephalitogenic lymphocyte–induced EAE	↑ TRAIL in active lymphocytes	Induce neuron apoptosis	Intracisternal application of sDR5	Decreased brain cell death	^106^
TRAIL/TRAIL‐R system play as a protect role					
Pneumococcal meningitis model of wild‐type and TRAIL −/− mice	/	TRAIL −/− mice with prolonged inflammation	Intrathecal application of rTRAIL	Limited excessive immune responses; decreased apoptosis	^85^
WNV meningitis model of wild‐type and TRAIL −/− mice	/	TRAIL −/− mice with defects in CD8 T cells and delayed viral clearance	Transfer of WNV‐primed wild‐type	CD8 T cells produced TRAIL to clear WNV infection	^84^
30 mins of tMCAO‐induced IPC	↑ decoy receptors; ↓ TRAIL and death receptors	Reduce inflammatory response; ameliorate ICS‐induced damage	Carry out IPC before ICS	IPC protected brain from forthcoming ICS by inducing decoy receptors	^49^
Tri‐(FAO‐FAR)–induced RPC	↑ decoy receptors; ↓ death receptors	Suppress extrinsic apoptosis; ameliorate ICS‐induced damage	Carry out RPC before ICS	RPC protected brain from forthcoming ICS by inducing decoy receptors	^54^
Myelin oligodendrocyte glycoprotein induced EAE	/	Inhibit autoimmune inflammation	Chronic TRAIL blockade with sDR5	Enhanced T cell responses and exacerbated EAE; Not regulate apoptosis of immune cells	104, 109
			rTRAIL	Delayed the onset and reduced the severity of EAE	^108^
			Intrathecal delivery of plasmid coding for OX40‐TRAIL	Decreased the severity of disease	^119^
			Treated with Fn14·TRAIL	Inhibited the clinical course of EAE	^120^

Abbreviation: 3 × Tg‐AD, triple transgenic mouse model of Alzheimer's disease; A‐MI/MA, active microglia and macrophages; BCCA, bilateral common carotid artery; CNS, central neural system; EAE, experimental autoimmune encephalomyelitis; FAO, femoral artery occlusion; FAR, femoral artery reperfusion; Fn14, a tumour necrosis factor–like weak inducer of apoptosis receptor; ICS, ischaemic cerebral stroke; IPC, ischaemic pre‐conditioning; OX40, a tumour necrosis factor receptor; RPC, remote limb pre‐conditioning; rTRAIL, recombinant TRAIL; SCI, spinal cord injury; sDR5, soluble DR5; tMCAO, transient middle cerebral artery occlusion; WNV, West Nile virus.

Additionally, to comprehensively elucidate the pathogenesis of diseases, it is important to combine new technologies such as intrathecal delivery of gene therapeutics to overcome limitations of CNS uptake of agents employed in EAE treatment or utilize TRAIL itself to increase the expression of decoy receptors or their affinity for TRAIL. Further studies should be performed to investigate the potential of TRAIL as a biomarker and reveal the precise molecular mechanisms of TRAIL. The clinical utility of TRAIL should be tested based on large sample–sized clinical cohort studies.

## CONFLICT OF INTEREST

The authors declare that they have no competing interests.

## AUTHOR CONTRIBUTION

**Shiqi Gao:** Resources (equal); Writing‐original draft (equal). **Yuan Fang:** Writing‐review & editing (equal). **Sheng Tu:** Resources (equal); Writing‐original draft (equal). **Huaijun Chen:** Writing‐review & editing (equal). **Anwen Shao:** Conceptualization (equal); Supervision (equal); Writing‐review & editing (equal).

## Data Availability

No data, models or code was generated or used during the study.
